# Comparison of the prognostic value of stromal tumor-infiltrating lymphocytes and CD3 + T cells between schistosomal and non-schistosomal colorectal cancer

**DOI:** 10.1186/s12957-023-02911-3

**Published:** 2023-02-01

**Authors:** Weixia Wang, Yingyi Zhang, Jican Liu, Hongyan Jing, Kui Lu, Limei Wang, Ting Zhu, Yanchao Xu, Dacheng Bu, Meihong Cheng, Jing Liu, Weidong Shen, Junxia Yao, Sinian Huang

**Affiliations:** Department of Pathology, Qingpu Branch of Zhongshan Hospital, Fudan University, Shanghai, 200032 People’s Republic of China

**Keywords:** Tumor-infiltrating lymphocytes, CD3 + cell, CD20 + cell, Colorectal cancer, Schistosomiasis, Prognosis

## Abstract

**Aim:**

To compare the prognostic value of tumor-infiltrating lymphocytes (TILs) and CD3 + cells and CD20 + cells between schistosomal colorectal cancer (SCRC) and non-schistosomal CRC (NSCRC).

**Background:**

Although schistosomiasis has been basically eliminated, it has not been completely extinction in China, and occasional outbreaks occur in Europe recently. The role of immune cells in the immune microenvironment of SCRC and NSCRC is remaining obscure, and the inflammation-based prognostic systems of SCRC has rarely been reported.

**Methods:**

HE-stained sections of 349 colorectal cancer (CRC) tumors, which were completely resected, were evaluated for density of TILs. Meanwhile, we evaluated CD3 + T lymphocytes and CD20 + B lymphocytes by immunochemistry. The relationship of these infiltrating immune cells with clinicopathological features, including schistosomiasis, and clinical outcomes was evaluated, and the prognostic roles of TILs in SCRC and NSCRC were explored.

**Results:**

Except for age (*P* < 0.0001), there were no significant differences between NSCRC and SCRC patients in clinicopathological features (*P* > 0.0*5*). Beside, the positive expression pattern of sTILs, iTILs, CD3, and CD20 between NSCRC and SCRC patients was also similar (*P* > 0.0*5*). In the whole cohort, sTILs and CD3 were defined as independent prognostic factors (*P* = 0.031 and *P* = 0.003, respectively). CD3 was an independent prognostic factor both in the NSCRC and SCRC set (*P* = 0.026 and *P* = 0.045, respectively). Higher sTILs, CD3, and CD20 were correlated with less aggressive tumor characteristics in the whole cohort and in subgroups.

**Conclusion:**

Although CD3 was an independent prognostic factor for both NSCRC and SCRC set, there were no significant differences between SCRC and NSCRC patients in sTILs, CD3, CD20, and in other clinicopathological features.

**Supplementary Information:**

The online version contains supplementary material available at 10.1186/s12957-023-02911-3.

## Introduction

Schistosomiasis is an infectious disease that affects more than 230 million people worldwide, according to conservative estimate [1]. In Asia, *Schistosoma japonicum* infection is considered as a risk factor for colorectal cancer. Qingpu District of Shanghai is used to be one of the 10 areas with serious schistosomiasis epidemic in China [2]. China has achieved considerable success in combating this disease, and the incidence and the prevalence of schistosomiasis has dropped [3]. However, problems of treatment and outcome of a large number of the late schistosomiasis patients left over from history still remain. In addition, multiple factors affect schistosomiasis epidemics, such as an increase in the number and spread of *Oncomelania* snails. Thus, there is still a risk of a rebound in the incidence in some areas [4]. In addition, schistosome eggs were occasionally found in pathological specimens of CRC under the microscope in our daily work.

Growing evidences have arisen in recent decades that inflammation is the cause of many malignant tumors [5, 6]. As the fourth most common cancer and the second leading cause of cancer deaths in the world [7], CRC represents an increasing number of cancers that correlated with inflammation [5, 8, 9]. Several studies have suggested that long-term inflammation caused by chronic schistosomal infection is a key factor in the carcinogenic process of CRC [10]. It was reported that the sequestered eggs in the mucosa and submucosa incite a severe focal inflammatory reaction with cellular infiltration and consequent minute mucosal ulcerations and microabscesses and granuloma formation. The continuous irritation produced by the egg nests eventually leads to fibrosis, mucosal hyperplasia, polyposis, and pseudopolyposis [11, 12]. However, all these studies were based on the analysis of data of clinicopathological characteristics; inflammation-based prognostic systems have been rarely reported in the literature. Besides, our previous study showed that schistosomiasis was an independent poor prognosis for CRC patients [13], and clinicopathological characteristics were different between CRC patients with schistosomiasis and without schistosomiasis [13]. But the evidence that chronic inflammatory process occurring in schistosomiasis plays an important role in CRC progression is not provided in the study.

The immune system is known to act against tumors, and it has been postulated that TILs reflect a tumor-related immune response [14–16]. TILs have been shown to provide prognostic and potentially predictive value in numerous literatures [16–18]. Accumulating evidence suggests that the extent of lymphocytic infiltration in tumor tissue can be assessed as a major parameter by evaluation of HE-stained tumor sections. K. M. Ropponen et al. evaluated TILs in the center and periphery of the tumors and around invasive carcinoma cells by HE-stained tumors sections, and their data showed that TILs can provide important prognostic information in colorectal cancer to be used in evaluating for adjuvant therapy in different tumor stages [19]. Ann C. Eriksen et al. reported that low CD3 + and CD8 + TILs were associated with inferior prognosis of stage 2 CRC by immunohistochemistry. Zhang et al. [20] showed that the presence of intratumoral CD3 + T cells was associated with improved survival in epithelial ovarian cancer. Yamei Zhao et al. combined the subtypes of TILs and the infiltrating sites with the anatomical sites of CRC to assess the association between each subset of TILs and the survival outcome by meta-analysis. Their results demonstrated that high-density TILs reflect favorable prognostic value in CRC [21]. Tumor-infiltrating CD20 + B cells have been associated with favorable outcomes in breast, cervical, and non-small cell lung cancer [22–24]. And Sofia Edin et al. showed that CD20 + B lymphocytes were associated with favorable survival in CRC [25]. Katy Milne et al. have shown that tumor-infiltrating CD20 + TILs were strongly associated with patient survival in high-grade serous ovarian cancer [26]. However, the relationship between schistosomiasis and these infiltrating immune cells, and the prognostic value of them in schistosomal CRC, has never been reported. In the present study, we undertook a study of TILs, CD3 + T, and CD20 + B cells to better determine the effect of schistosomiasis on CRC patients’ outcomes and to compare the prognostic role of infiltrating immune cells in non-schistosomal and schistosomal CRC.

## Materials and methods

### Patients and samples

A total of 349 CRC patients were enrolled in this retrospective study after applying the following inclusion/exclusion criteria. All patients had received curative resection without preoperative chemotherapy at Qingpu Branch of Zhongshan Hospital affiliated to Fudan University, from January 2008 to August 2016. And all of operations followed the principle: adequate resection margins, en bloc high ligation of the inferior mesenteric artery (IMA), and lymphadenectomy. All circumferential margins were cleared. The number of positive lymph nodes and total number of retrieved lymph nodes were recorded. The inpatient medical records and pathological reports were reviewed, and the patients were followed up by telephone. OS is defined as the interval from the surgical operation date to the last follow-up or death caused by CRC. Inclusion criteria included the following: (i) patients with CRC as primary focus, (ii) none of these patients had received any prior anti-tumor therapy, and (iii) patients were diagnosed as adenocarcinoma by pathology after resection of CRC. Exclusion criteria included the following: (i) Tis tumors; (ii) patients who lacked complete information; (iii) patients with synchronous malignancy, such as liver cancer, lung cancer, and ovarian cancer, were excluded; and (iv) patients with survival time less than 1 month. Two expert pathologists reviewed HE-stained slides to determine the diagnosis and to restage the tumors according to the eighth edition of American Joint Committee on Cancer (AJCC).

### Ethics approval and consent to participate

This study was approved by the medical ethics committee of Fudan University, in accordance with the Helsinki Declaration of 1975. Prior written informed consent was obtained from all patients.

### Detection of schistosome ova

Schistosome ova were observed in all of original HE-stained formalin-fixed paraffin-embedded (FFPE) sections (usually 4–6 slides), which were examined at × 10 and × 40 magnification fields using a conventional light microscope by two pathologists who were blinded to clinic data. The diagnosis of schistosomiasis was done by finding schistosome eggs in anywhere in the HE-stained slides. Patients with schistosomal CRC were identified by the presence of *Schistosoma* eggs in the intestinal tissues.

### Assessment of tumor budding

Tumor budding was defined as the presence of dedifferentiated single cells or small clusters of up to 5 cells at the invasive front of CRC [27]. To assess tumor budding in the 10-HPF method [28], the invasive front is first scanned at low magnification (× 4– × 10) to identify areas of highest budding density. Tumor buds are then counted under high magnification (× 20), and the tumor budding count is reported. The evaluation of tumor budding was conducted by two pathologists who were blinded to clinic data. Five tumor budding counts were used as breakthrough point. In brief, tumor bud counts greater than or equal to 5 were defined as high group, otherwise as low group.

### Histologic evaluation of TIL

Evaluation of TILs was performed as previously described [29]. TILs were performed in H&E-stained FFPE sections, which were examined at × 10 and × 40 magnification fields using a conventional light microscope by two pathologists. Intratumoral-infiltrating lymphocytes (iTILs) were defined as the percentage of mononuclear cells within the epithelium of the invasive tumor cell nests. Stromal-infiltrating lymphocytes (sTILs) were defined as the percentage of tumor stroma containing infiltrating lymphocytes (area occupied by mononuclear cells in tumor stroma/total stromal area). Here, in our study, more than 2% of either stromal or intratumoral TILs were defined as TILs rich. The mean value was used for the analyses presented. Neither pathologist had any knowledge of the clinical information.

### Immunohistochemical analysis

Three- to 5-μm-thick CRC tissues were consecutively cut and subsequently dewaxed and rehydrated through graded alcohols. Slides were immunohistochemically stained in Roche Ventana BenchMark XT automated slide stainer (Ventana Medical Systems, Roche, France) according to the manufacturer’s instructions. Monoclonal and polyclonal antihuman antibodies were used for identification of CD3 + T cells (anti-CD3, NCL-L-CD3-565, Dako), and CD20 + TILs (anti-CD20, Ab4055, Abcam). Quantitative evaluation of CD3 + and CD20 + TILs was performed in 10 most representative high-power fields (magnification × 40) per tissue section using a Leica DM2000 microscope (Leica Co., Germany). The results were carried out blindly to the clinical data.

Evaluation of T-cell marker density was carried out blinded to clinicopathologic information. Individual cores were examined by two observers and annotated to ensure that only normal colonic epithelium or viable tumor tissue was included in the area of analysis. No attempt was made to evaluate the various tumor compartments separately (e.g., stroma, tumor cell nests). The whole section slides were observed under low magnification × 20. Then each tissue is divided into four quadrants, and the percentage of CD3 and CD20-positive cells in the four quadrants is calculated in the unit of the number of positive cells/mm^2^, and the average value of the four fields is utilized.

### Statistical analysis

Data were analyzed using SPSS (version 20.0; IBM Corp.) and GraphPad 5.0. Fisher’s exact and chi-square tests were utilized to perform correlation between clinicopathological characteristics and TILs and schistosomiasis. The main reasons are as follows: Firstly, there is more than 300 cases enrolled in the cohort, and the sample size is large enough. Secondly, the theoretical frequency T in each grid is greater than 5. Thirdly, some indexes are continuous variables, and some are noncontinuous variables, which are converted into categorical variables. K-M curves with log-rank tests were used to determine the prognostic significance for OS. Every variable was analyzed using univariate analysis to identify all potentially important predictors, and then, variables with *P* ≤ 0.05 in the univariate analysis were included in a multivariate analysis. Finally, multivariate Cox regression analysis was performed to identify predictive factors for OS. Clinically relevant variables that may have impacted outcomes are as follows: age, gender, TNM stage, lymph node metastasis, and histological type. *P* < 0.05 was considered to indicate a statistically significant difference.

## Results

### Study patients

**Fig. 1:**
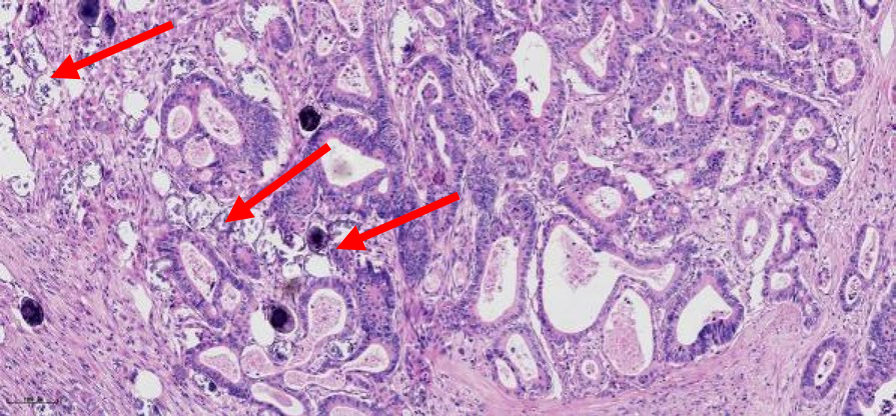
Typical sample of schistosomiasis-associated colorectal cancer; the red arrows indicate schistosome ova (HE, × 200)

Patient characteristics are summarized in Table 1. In the whole cohort, the age of patients at diagnosis ranged from 33 to 91 years (median age was 69 years) and were predominantly male (60.7%, 212 out of 349). About 39% (137 out of 349) CRC patients were infected with *Schistosoma* (Fig. 1). By anatomic site, 26.9% tumors were in the rectum, 33.0% in left colon, and 40.1% in right colon. Patients at late-stage disease were 45.6%. Patients without lymph node metastasis were 58.7%. On the basis of the AJCC Staging Manual (eighth edition), there were very few highly differentiated cases in the follow-up data. Thus, highly differentiated and moderately differentiated cases were classified as “well differentiation,” and poorly differentiated cases were classified as “poor differentiation.” Patients with well-differentiated tumor were 75.9%.

**Table 1:** Clinicopathological characteristics of colorectal cancer cohort

Characteristics	All patients (*N* = 349)	
	*N*	*%*
Age (< 60 years)	82	*23.5*
Gender (male)	212	*60.7*
Tumor location		
Rectum	94	*26.9*
Left colon	115	*33.0*
Right colon	140	*40.1*
Tumor size (< 5 cm)	173	*49.6*
Tumor differentiation		
Well/moderately diff	265	*75.9*
Poorly diff	84	*24.1*
Lymphangio (positive)	125	*35.8*
Nervous invasion (positive)	32	*9.2*
Tumor deposit (> 2)	42	*12.0*
Colonic perforation (yes)	13	*3.7*
Tumor budding (≥ 5 cells)	212	*60.7*
Ulceration (yes)	149	*42.7*
Histological type		
Adenocarcinoma	306	*87.7*
Mucinous/signet ring cell carcinoma	43	*12.3*
Pathological T stage		
T1-2	83	23.8
T3-4	266	76.2
Lymph node metastasis		
No	205	*58.7*
Yes	144	*41.3*
TNM stage		
I + II	190	*54.4*
III + IV	159	*45.6*
*Schistosomiasis*	137	*39.3*

### Comparison of clinicopathological features and infiltrating immune cells between NSCRC and SCRC patients

Patients were divided into two groups: NSCRC and SCRC set based on the status of schistosomal infection. The comparison of clinicopathological features and infiltrating immune cells between NSCRC and SCRC patients was performed. Except for age (Table 2 and Supplementary Fig. 1, *P* < 0.0001), there were no significant differences between SCRC and NSCRC patients in clinicopathological features (Table 2, *P* > 0.05). Unexpectedly, there were also no significant differences between the two groups in sTILs, iTILs, CD3, and CD20 (Table 2, *P* > 0.0*5*).

**Table 2:** The association between schistosomiasis and clinicopathological features and infiltrating immune cells

Characteristics	NSCRC (*N* = 188*)	SCRC (*N* = 126*)	*p*
**Age (**< **60 years)**			< 0.0001
< 60	68	5	
≥ 60	120	121	
**Gender**			0.348
Male	79	46	
Female	109	80	
**Tumor site**			0.530
Rectum	47	36	
Left colon	59	43	
Right colon	82	47	
**Tumor size**			0.908
< 5 cm	95	62	
≥ 5 cm	93	64	
**Tumor differentiation**			0.500
Well/moderately diff	146	93	
Poorly diff	42	33	
**Lymphangio**			0.811
Negative	119	82	
Positive	69	44	
**Nervous invasion**			1.000
Negative	171	115	
Positive	17	11	
**Tumor deposit**			1.000
≤ 2	165	110	
> 2	23	16	
**Colonic perforation**			1.000
No	181	121	
Yes	7	5	
**Ulceration**			0.559
**No**	113	71	
**Yes**	75	55	
**Pathological T stage**			0.689
T1-2	48	29	
T3-4	140	97	
**LNM**			0.907
No	108	74	
Yes	80	52	
**TNM stage**			0.730
I + II	99	69	
III + IV	89	57	
**Tumor budding**			0.906
< 5 cells	75	49	
≥ 5 cells	113	77	
**Histological type**			0.866
Adenocarcinoma	164	109	
Mucinous/SRCC	24	17	
**sTILs**			0.287
Poor	67	53	
Rich	121	73	
**iTILs**			0.164
Poor	185	120	
Rich	3	6	
**CD3**			0.219
Poor	123	91	
Rich	65	35	
**CD20**			0.728
Poor	104	73	
Rich	84	53	

Abbreviations: *NSCRC* Non-schistosomal colorectal cancer, *SCRC* Schistosomal colorectal cancer, *N* Number, *LNM* Lymph node metastasis, *SRCC* Signet ring cell carcinoma, *sTILs* stromal tumor-infiltrating lymphocytes, *iTILs* intratumoral-infiltrating lymphocytes. The association between schistosomiasis and clinicopathological characteristics was evaluated by using the chi-square and Fisher’s exact tests

^*^Although 349 CRC patients were enrolled in the cohort, some sample tissues were unavailable for the immunohistochemical assay, such as those with high-fat content. Finally, there were 314 cases could be used for the following analysis of CD3 and CD20

The correlation between sTILs and CD3 and CD20 and clinicopathological features was shown in Supplementary Table 1. The results showed that higher sTILs infiltration was correlated with smaller tumor size (*P* < 0.001), less deeper pathological T stage (*P* < 0.001), well/moderately differentiated tumors (*P* = 0.034), and less number of tumor budding (*P* < 0.001), which is an important additional prognostic factor for patients with CRC [27, 28]. Besides, higher sTILs were also correlated with higher iTILs (*P* = 0.016), CD3 (*P* < 0.001), and CD20 (*P* = 0.019). As shown in Supplementary Table 1, higher CD3 were correlated with smaller tumor size (*P* = 0.011), less number of tumor budding (*P* = 0.013), earlier TNM stage (*P* < 0.001), and less deeper pathological T stage (*P* = 0.010). CD3 was also positively correlated with CD20 (*P* = 0.001). There was no association between CD20 and clinicopathological features.

### Univariate and multivariate regression analysis

**Fig. 2:**
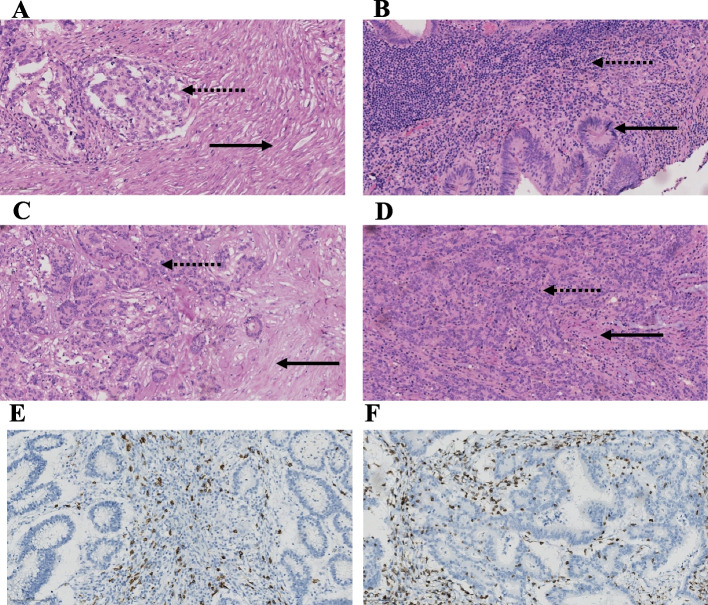
Representative views of TILs and CD3 and CD20 in CRC. Examples of low TILs (**A**) and high TILs (**B**) in stromal compartments (sTILs) (HE, × 200) and low TILs (**C**) and high TILs (**D**) in intraepithelial compartments (iTILs) (HE, × 200), dashed arrows and solid arrows indicate tumoral and stromal area. Representative images of CD3 + (**E**, × 200) and CD8 + (**F**, × 200)

Representative infiltrating immune cells staining is shown in Fig. 2. In the whole cohort, the optimum cutoff value of TILs and CD3 and CD20 was determined by X-tile program. Accordingly, stromal and intratumoral TILs ≥ 2% was defined as TILs rich, otherwise as TILs poor, and sTILs rich were observed in 62.8% cases (219 out of 349), iTILs rich was observed in 2.6% cases (9 out of 349). Some FFPE tissues with high-fat content were unavailable for IHC assay. Finally, 314 samples were suitable for following analysis. CD3 ≥ 24% was defined as CD3 rich, otherwise as CD3 poor, and CD3 rich were observed in 31.8% cases (100 out of 314). In addition, CD20 ≥ 4% was defined as CD20-rich, otherwise as CD20-poor, and CD20-rich were observed in 43.6% cases (137 out of 314). In the whole cohort, multivariate Cox regression analysis identified sTILs (*P* = 0.031) and CD3 (*P* = 0.003) as independent prognostic factors as shown in Table 3. However, iTILs and CD20 were not independent prognostic factors. In addition, some clinical factors that independently associated with OS (Table 2) were gender (*P* = 0.001), TNM stage (*P* < 0.0001), differentiation (*P* = 0.039), lymph vascular invasion (*P* = 0.012), and tumor deposit (*P* < 0.0001).

**Table 3:** Univariate and multivariate Cox regression of clinicopathological features and infiltrating immune cells for overall survival

Variable	All patients (*N* = 314)		NSCRC (*N* = 188)		SCRC (*N* = 126)	
	*p*	HR (95% *CI*)	*p*	HR (95% *CI*)	*p*	HR (95% *CI*)
**Univariate analysis**						
sTILs	0.000	0.553 (0.398–0.769)	0.002	0.499 (0.321–0.776)	0.162	0.704 (0.431–1.151)
iTILs	0.497	1.413 (0.521–3.831)	0.949	1.047 (0.256–4.281)	0.201	2.533 (0.609–10.546)
CD3*	0.001	0.478 (0.307–0.745)	0.012	0.518 (0.309–0.868)	0.014	0.318 (0.127–0.796)
CD20*	0.007	0.613 (0.428–0.877)	0.029	0.600 (0.379–0.950)	0.044	0.573 (0.333–0.985)
Age (< 60 years)	0.013	1.731 (1.124–2.666)	0.156	1.410 (0.877–2.268)	0.232	21.827 (0.139–3436.270)
Gender (male/female)	0.013	1.562 (1.100–2.216)	0.022	1.738 (1.082–2.790)	0.307	1.311 (0.779–2.207)
Tumor size (5 cm)	0.834	1.036 (0.746–1.437)	0.526	0.864 (0.550–1.357)	0.320	1.282 (0.786–2.089)
Tumor site						
Rectum		Refer		Refer		Refer
Left colon	0.908	1.025 (0.676–1.553)	0.677	0.891 (0.516–1.537)	0.539	1.229 (0.636–2.376)
Right colon	0.501	0.869 (0.578–1.307)	0.054	0.587 (0.341–1.010)	0.128	1.636 (0.867–3.084)
Pathological T stage	0.000	2.596 (1.565–4.307)	0.001	3.375 (1.625–7.008)	0.087	1.851 (0.915–3.747)
Lymph node metastasis	0.000	2.793 (2.002–3.895)	0.000	2.469 (1.581–3.856)	0.000	3.552 (2.141–5.894)
TNM stage	0.000	3.216 (2.279–4.537)	0.000	2.797 (1.765–4.434)	0.000	4.219 (2.479–7.128)
Tumor differentiation	0.000	1.879 (1.326–2.662)	0.003	2.013 (1.260–3.216)	0.054	1.668 (0.991–2.809)
Lymphangio	0.000	2.038 (1.467–2.832)	0.000	2.829 (1.811–4.419)	0.275	1.321 (0.801–2.180)
Nervous invasion	0.143	1.492 (0.873–2.552)	0.327	1.417 (0.706–2.844)	0.500	1.727 (0.741–4.024)
Tumor deposit	0.000	3.979 (2.667–5.936)	0.000	3.967 (2.353–6.688)	0.000	4.138 (2.205–7.769)
Colonic perforation	0.813	0.887 (0.328–2.397)	0.767	1.190 (0.375–3.670)	0.508	0.506 (0.070–3.657)
Tumor budding (≤ 5 cells)	0.001	1.877 (1.297–2.715)	0.000	2.500 (1.492–4.188)	0.421	1.247 (0.729–2.134)
Schistosomiasis	0.049	1.390 (1.001–1.929)	—	—	—	—
Ulceration	0.617	0.919 (0.659–1.281)	0.743	1.077 (0.690–1.681)	0.212	0.725 (0.437–1.201)
Histological type	0.705	1.096 (0.683–1.757)	0.280	1.403 (0.759–2.594)	0.467	0.760 (0.362–1.594)
**Multivariate analysis**						
Gender	0.001	1.886 (1.295–2.746)	0.021	1.812 (1.093–3.004)	—	
TNM stage	0.000	2.099 (1.403–3.139)	—	—	0.000	3.703 (2.149–6.381)
Tumor budding (≤ 5 cells)	—	—	0.050	1.647 (0.988–2.745)	—	—
Tumor differentiation	0.039	1.499 (1.021–2.203)	0.021	1.826 (1.095–3.044)	—	
CD3*	0.003	0.481 (0.297–0.781)	0.026	0.525 (0.297–0.927)	0.045	0.404 (0.160–1.021)
sTILs	0.031	0.660 (0.452–0.963)	0.034	0.570 (0.339–0.959)	—	—
Lymphangio	0.012	1.580 (1.106–2.256)	0.006	1.994 (1.223–3.252)	—	—
Tumor deposit	0.000	2.818 (1.743–4.555)	0.000	3.837 (2.053–7.170)	—	—

—Data is non-significant; Abbreviation: *NSCRC* Non-schistosomal colorectal cancer, *SCRC* Schistosomal colorectal cancer, *HR* Hazard ratio, *CI* Confidence interval, *sTILs* stromal tumor-infiltrating lymphocytes, *iTILs* intratumoral-infiltrating lymphocytes, *P* < 0.05 was defined as the criterion for variable deletion when performing backward stepwise selection

^*^Missing data (although 349 CRC patients were enrolled in the cohort, some sample tissues were unavailable for the immunohistochemical assay, such as those with high-fat content. Finally, there were 314 cases could be used for the following analysis of CD3 and CD20)

Further analysis was conducted to explore the prognostic significance of TILs in patients with and without *schistosomiasis*, respectively. The optimum cutoff value of sTILs, iTILs, CD3, and CD20 in NSCRC and SCRC was also determined by X-tile program. In the NSCRC group, gender, sTILs, CD3, CD20, pathological T stage, lymph node metastasis, TNM stage, tumor budding, tumor differentiation, lymph vascular invasion, tumor budding, and tumor deposit were significant prognostic factors for OS (*P* < 0.05) as shown in Table 3. When variables with *P* ≤ 0.05 in the univariate analysis were included in a multivariate analysis, CD3 (*P* = 0.026), sTILs (*P* = 0.034), gender (*P* = 0.021), tumor budding (*P* = 0.050), tumor differentiation (*P* = 0.021), lymph vascular invasion (*P* = 0.006), and tumor deposit (*P* < 0.001) were independent prognostic factors in the NSCRC group. However, merely TNM stage (*P* < 0.0001) and CD3 (*P* = 0.045) were independent predictors in SCRC group (Table 3).

### Survival analysis

Given that iTILs were not associated with OS in the univariate and multivariate Cox regression analysis, the K-M curves with log-rank tests of iTILs were ignored. To investigate the prognostic value between clinical outcomes and sTILs, and CD3 and CD20, Kaplan–Meier analysis was conducted in the total cohort according to percentage of sTILs, CD3, and CD20. Mean and median time to OS were 68 and 69 (1.25–134.39) months, respectively. During the follow-up, there were 42.6% (134 out of 314) patients who died.

**Fig. 3:**
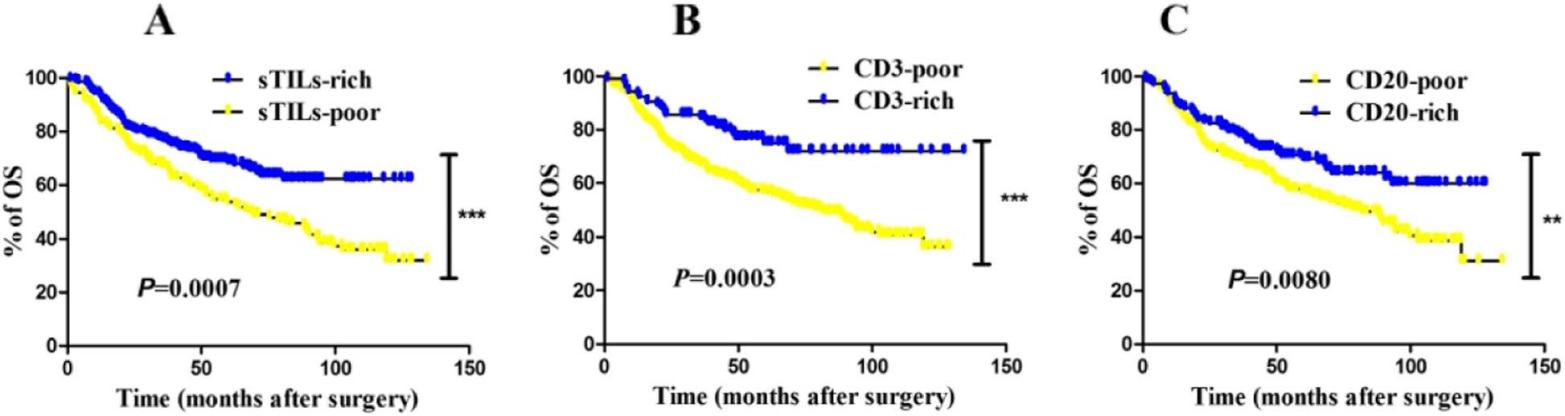
Kaplan–Meier curves of overall survival (OS) revealing prognostic significance of TILs in the whole cohort. Among 349 patients, a significantly better prognosis was observed in CRC patients with higher sTILs (≥ 2%) (*P* = 0.0007) **A**. Although 349 CRC patients were enrolled in the cohort, but some sample tissues were unavailable for the immunohistochemical assay, such as those with high-fat content. Finally, there were 314 cases could be used for the following analysis of CD3 and CD20. Results showed higher CD3-positive expression **B** (*P* = 0.0003) and higher CD20-positive expression **C** (*P* = 0.0080)

Patients with sTILs rich gained significant survival benefit compared with those with sTILs poor (Fig. 3A; *P* = 0.0007). Meanwhile, patients with CD3 rich were associated with favorable OS compared with that of with CD3 poor (Fig. 3B; *P* = 0.0003). Besides, patients with CD20 rich were also associated with favorable OS compared with those of CD20 poor (Fig. 3C; *P* = 0.0080).

**Fig. 4:**
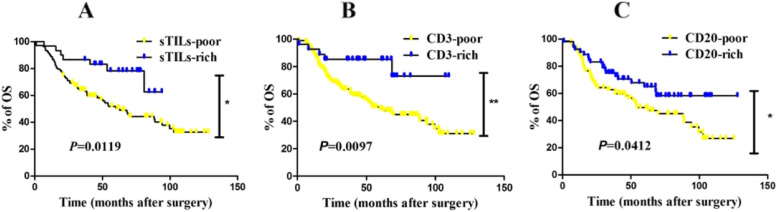
Kaplan–Meier curves of overall survival (OS) revealing prognostic significance of TILs in CRC patients with schistosomiasis (*N* = 126). There was significantly association between OS and sTILs (≥ 2% was defined as sTILs-rich group, otherwise as sTILs-poor group) (*P* = 0.0119) **A**, higher CD3-positive expression **B** (*P* = 0.0097), and higher CD20-positive expression (*P* = 0.0412) **C** was associated with better prognosis in CRC patients with schistosomiasis. Note: Although 349 CRC patients were enrolled in the cohort and used for HE staining of TILs, but some sample tissues were unavailable for the immunohistochemical assay, such as those with high-fat content. Finally, there were 314 cases could be used for the following analysis of CD3 and CD20. Among these 314 cases, the number of patients without schistosomiasis was 126

**Fig. 5:**
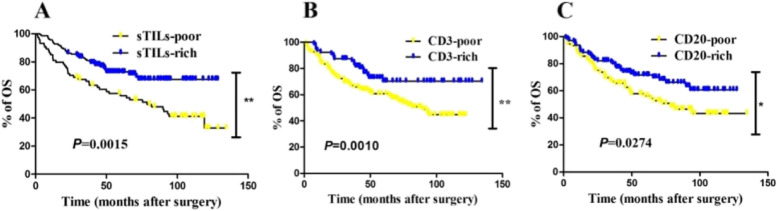
Kaplan–Meier curves of overall survival (OS) revealing prognostic significance of TILs in CRC patients without schistosomiasis (*N* = 188). There was significantly association between OS and sTILs (≥ 2% was defined as sTILs-rich group, otherwise as sTILs-poor group) (*P* = 0.0015) **A**, higher CD3-positive expression **B** (*P* = 0.0010), and higher CD20-positive expression (*P* = 0.0274) **C** was associated with better prognosis in CRC patients without schistosomiasis. Note: Although 349 CRC patients were enrolled in the cohort and used for HE staining of TILs, but some sample tissues were unavailable for the immunohistochemical assay, such as those with high-fat content. Finally, there were 314 cases could be used for the following analysis of CD3 and CD20. Among these 314 cases, the number of patients with schistosomiasis was 188

Considering that schistosomiasis was a risk factor in the univariate analysis as shown in Table 3, patients were further stratified by status of schistosomal infection. Higher sTILs and CD3 and CD20 were significantly associated with better prognosis compared with lower sTILs and CD3 and CD20 in the SCRC groups (Fig. 4; *P* = 0.0119, *P* = 0097, and *P* = 0.0412, respectively). In the NSCRC group, higher sTILs and CD3 and CD20 were also significantly associated with favorable OS (Fig. 5; *P* = 0.0015, *P* = 0010, and *P* = 0.0274, respectively).

## Discussion

Schistosomiasis is a common disease worldwide. In endemic areas, schistosomal infestation has been implicated in the etiology of several human malignancies including the bladder, liver, and CRC [30, 31]. Ye et al. [32] reported that intestinal schistosomiasis was a risk factor for CRC, and that the lesions caused by the disease might be considered precancerous. Our previous study demonstrated that schistosomiasis was an independent worse prognosis for OS in CRC patients [13]. In this study, we further confirmed that schistosomiasis was an unbeneficial prognosis factor for OS in CRC patients. However, sufficient evidence supports a causal relationship between schistosomal infection, and CRC has apparently low status within the canons of medicine and reports from the publishing world [33].

Specimens from patients with schistosomiasis showed associated inflammatory changes, pseudopolyps, and transitional mucosal changes of schistosomal granulomatous disease progressing to mucosal atypia and to carcinoma were reminiscent of colorectal carcinoma in patients with ulcerative colitis [34]. It was known that TILs reflect an active inflammatory tumor microenvironment. Accordingly, it was hypothesized that schistosomiasis infection could associate with vigorous TILs infiltration. In this study, we found that sTILs were an independent favorable predictor factor for OS in the whole cohort. This was consistent with previous studies [18, 35, 36]. Stromal TILs were also an independent favorable predictor factor in patients without schistosomiasis, but was not in patients with schistosomiasis. These results seem inconsistent with expectations and assumptions. It was reported that aging lead to immunosenescence [37]. We found that patients with schistosomiasis were obviously older than patients without schistosomiasis. We wonder whether immune response was inert in patients with schistosomiasis. Thus, patients were restratified by age, and results showed that sTILs were associated with favorable OS in NSCRC patients but not in SCRC, regardless of age (Supplementary Fig. 2). This might be related to the complex immune response and immune microenvironment caused by chronically schistosomal infection, and further work is needed to reveal the underlying mechanism. Additionally, comparative analysis of sTILs infiltration with clinicopathologic features revealed that higher sTILs infiltration correlated with less aggressive features of tumors. This result is consistent with the literatures [19, 38]. These results further confirmed that sTILs played crucial role in the development and progression of CRC.

In present study, we evaluated iTILs and sTILs, respectively. However, we found that iTILs are typically present in lower numbers and detected in fewer cases; they are more heterogeneous and are uneasy to observe on H&E-stained slides [29]. The percentage of iTILs of almost 98% cases in the total cohort was under 2%. This may help to explain that there was no association between iTILs and OS. Moreover, most current studies have found stromal TILs to be a superior and more reproducible parameter [39].

Given the functional heterogeneity of intratumoral lymphocytes, and negative immune regulators are present as part of a normal feedback loop reacting to an active and ongoing antitumor immune response, a focused evaluation of individual subsets may have limited value. Besides, the degree of lymphocytic infiltration assessed by simple evaluation of hematoxylin and eosin (H&E)-stained tumor sections has been shown to have predictive and prognostic value despite a lack of detailed information on the immune subpopulations of the infiltrate [35, 40–42].

CD20 + B cells form a major proportion of tumor-infiltrating lymphocytes in a variety of cancers [43–45]. There is currently a lack of consensus regarding the role of B cells in carcinogenesis and progression of solid tumors [46]. There is increasing evidence that lymphocyte infiltration correlates with improved response to chemotherapy in solid tumors [47–50]. Sofia Edin and colleague’s study supports a favorable prognostic value of tumor-infiltrating CD20 + B lymphocytes in CRC [51]. In our study, CD20 was associated with favorable OS in the univariate analysis in the whole cohort and in NSCRC and in SCRC subset, but it was not remained significance in multivariable analysis in none of the groups. This could be explained by the phenotypical and functional diversity of B-cell subtypes. The complex role of B cells in carcinogenesis is conceivably complex. There are many factors at play, which may influence the role of B cells in solid tumors, including tumor type and stage, the location, subset, and activation status of B cells, as well as other immune cells present within the tumor microenvironment [52].

The activation of T cells requires the T-cell receptor complex, which includes the co-receptor CD3 [53]. Our findings showed that both schistosomal and non-schistosomal CRC featured a high expression of CD3 + T cells that was significantly correlated with improved survival and acts as an independent prognostic indicator. Previous studies have shown that the presence of CD3 + T cells in the tumor microenvironment is related to improved prognosis in a wide range of tumors [54, 55]. Supporting our findings, recent studies have reported the abundance of CD3 + T cells in the tumor microenvironment as an independent prognostic factor for CRC, improving OS and recurrence-free survival [54, 55]. Thus, the high expression of CD3 + cells is likely reflecting immunocompetent T lymphocytes with anti-tumor activity and also highlights its potential roles in chronic inflammatory reaction caused by schistosomiasis. In addition, the results demonstrated that CD3 could be used as an accurate prognostic marker and for the selection of patient candidates for therapies targeting T lymphocytes, especially in schistosomal CRC patients.

Several limitations associated with the present study warrant mentioning. Firstly, the diagnosis of schistosomiasis was done by finding schistosome eggs in HE-stained slides, lacking of other methods to prove schistosome infection. This may lead to the missing of the number of schistosomiasis-positive cases. In order to reduce this limitation, schistosome ova were observed in all original HE-stained slides (usually 4–6 slides per case), which were obtained from original individual paraffin blocks and used for routine diagnosis. Besides, the medical records of the selected cases were checked carefully to further screen schistosome-positive reports. Secondly, the working group states that evaluating sTIL as a continuous variable will allow for more accurate statistical analysis [29], but in practice, most pathologists will not report specific values. Simplicity is needed for a pathological methodology to be accepted widely. For this reason, sTILs and iTILs were evaluated as a noncontinuous variable in this study. In the study, we found a positive correlation between sTILs and CRC outcomes, the precise functional roles of sTILs infiltration in CRC progression, and its underlying molecular mechanisms remain obscure. Thirdly, the immunohistochemical (IHC) staining of CD3 and CD20 was performed on discontinuous slides, and we could not perform multiplexed staining on the same slide, so we did not measure the positive ratio or raw number of CD3 + /CD20 + cells per TILs.

In summary, the results of this study identified CD3 + cell, but not TILs and CD20 + cell as an independent prognostic indicator for schistosomal and non-schistosomal CRC patients. And the clinicopathological features and positive expression pattern of these infiltrating immune cells between NSCRC and SCRC patients were similar. However, further work to confirm the prognostic role of CD3 in a larger cohort or in multi-organization is needed in further study.

## Supplementary Information

**Additional file 1:** **Supplementary Fig. 1.** The relationship between schistosomial infection and age distribution. The association was evaluated by using Kruskal-Wallis statistic. **Supplementary Fig. 2.** Kaplan-Meier curves of Overall Survival (OS) revealing prognostic significance of sTILs in CRC patients with different age group. **Supplementary Table. 1.** The association between clinicopathological characteristics and* schistosomiasis* and infiltrating immune cells.

## Data Availability

The datasets used and/or analyzed during the current study are available from the corresponding authors on reasonable request.

## Abbreviations

TILs: Tumor-infiltrating lymphocytes
CRC: Colorectal cancer
H&E: Hematoxylin and eosin
iTILs: Intratumor TILs
sTILs: Stromal TILs
SCRC: Schistosomal colorectal cancer
NSCRC: Non-schistosomal colorectal cancer
OS: Overall survival
IMA: Inferior mesenteric artery
AJCC: American joint committee on cancer
K-M: Kaplan–Meier
FFPE: Formalin-fixed paraffin embedded
