# Supplementary material for: Comparison of the prognostic value of stromal tumor-infiltrating lymphocytes and CD3 + T cells between schistosomal and non-schistosomal colorectal cancer
Source: World J Surg Oncol. 2023 Feb 1;21:31. doi: 10.1186/s12957-023-02911-3 (PMC9890788; doi:10.1186/s12957-023-02911-3)
Supplement: Supplementary file 1 — Additional file 1: Supplementary Fig. 1. The relationship between schistosomial infection and age distribution. The association was evaluated by using Kruskal-Wallis statistic. Supplementary Fig. 2. Kaplan-Meier curves of Overall Survival (OS) revealing prognostic significance of sTILs in CRC patients with different age group. Supplementary Table. 1. The association between clinicopathological characteristics and schistosomiasis and infiltrating immune cells. [file 12957_2023_2911_MOESM1_ESM.docx]

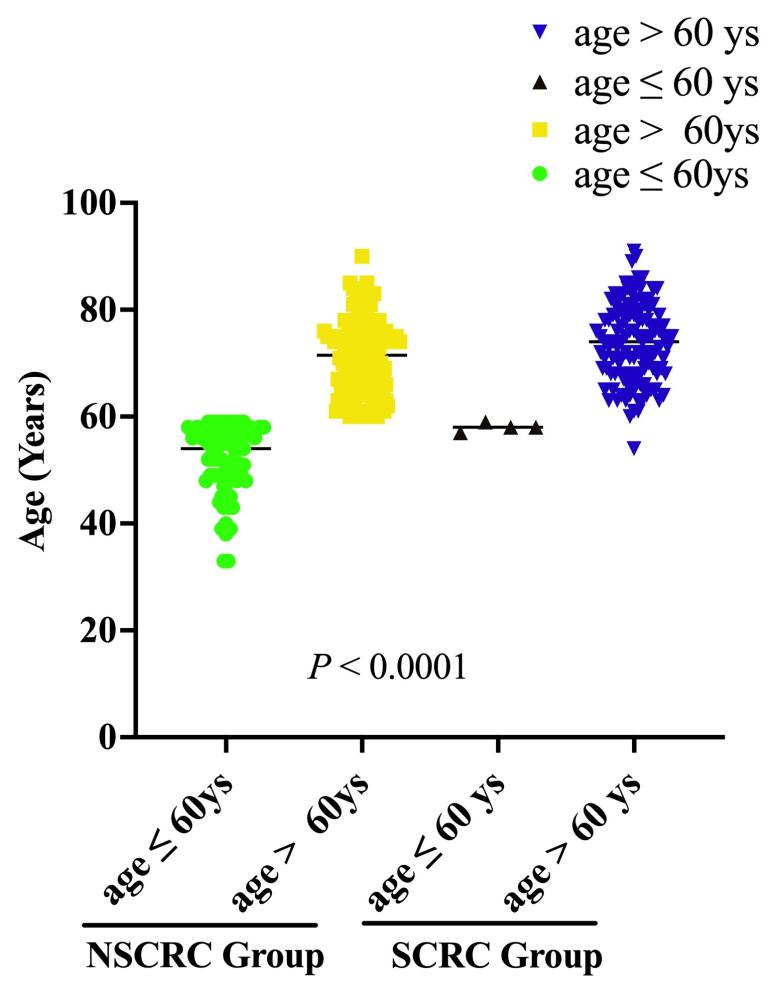


**Supplementary Fig.1** The relationship between schistosomial infection and age distribution. The association was evaluated by using Kruskal-Wallis statistic.


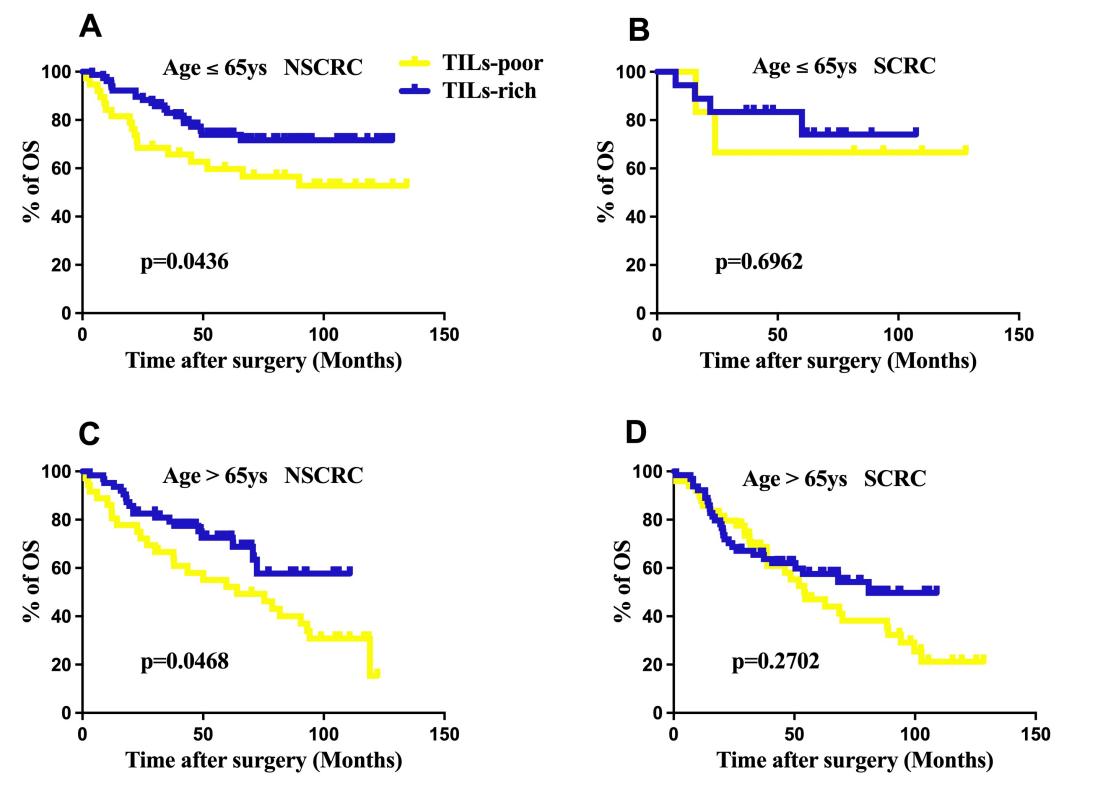


**Supplementary Fig.2 Kaplan-Meier curves of Overall Survival (OS) revealing prognostic significance of sTILs in CRC patients with different age group.** In the whole cohort, patients were stratified by age: patients younger than or equal to 65 years old and older than 65 years old. Stromal TILs associated with longer OS in the NSCRC patients who were younger than or equal to 65 years old (**A**) (*P*=0.0436). However, there were no significant difference between sTILs and OS in the SCRC patients of this age group (*P* =0.6962) (**B**); similarly, sTILs were associated with OS in NSCRC group (*P* =0.0468) (**C**); but not in SCRC patients who were older than 65 years old (*P* =0.2702) (**D**).

| **SupplementaryTable.1.** The association between clinicopathological characteristics and *schistosomiasis* and infiltrating immune cells | | | | | | | | | |
| --- | --- | --- | --- | --- | --- | --- | --- | --- | --- |
| Characteristic | sTILs | | *P* | CD3^*^ | | *P* | CD20^*^ | | *P* |
|  | Poor (≤2)  (N=130) | Rich (≥3%)  (N=219) |  | Poor (≤24)  (N=214) | Rich (≥25)  (N=100) |  | Poor (≤3)  (N=177) | Rich (≥4)  (N=137) |  |
| **Age(＜60ys)** |  |  | 0.498 |  |  | 0.775 |  |  | 0.060 |
| ＜60 | 30 | 52 |  | 51 | 22 |  | 34 | 39 |  |
| ≥60 | 100 | 167 |  | 163 | 78 |  | 143 | 98 |  |
| **Gender** |  |  | 0.070 |  |  | 0.711 |  |  | 0.564 |
| Male | 87 | 124 |  | 127 | 62 |  | 104 | 85 |  |
| Female | 43 | 95 |  | 87 | 38 |  | 73 | 52 |  |
| **Tumor site** |  |  | 0.110 |  |  | 0.085 |  |  | 0.115 |
| Rectum | 27 | 67 |  | 50 | 33 |  | 40 | 43 |  |
| Left colon | 49 | 66 |  | 77 | 25 |  | 58 | 44 |  |
| Right colon | 54 | 86 |  | 87 | 42 |  | 79 | 50 |  |
| **Tumor size** |  |  | ＜0.001 |  |  | 0.011 |  |  | 0.998 |
| ＜5cm | 49 | 124 |  | 96 | 61 |  | 88 | 69 |  |
| ≥5cm | 81 | 95 |  | 118 | 39 |  | 89 | 68 |  |
| **Tumor differentiation** |  |  | 0.034 |  |  | 0.321 |  |  | 0.183 |
| Well/moderately diff. | 113 | 170 |  | 159 | 80 |  | 140 | 99 |  |
| Poorly diff. | 17 | 49 |  | 55 | 20 |  | 37 | 38 |  |
| **Lymphangio** |  |  | 0.422 |  |  | 0.316 |  |  | 0.406 |
| Negative | 87 | 137 |  | 141 | 60 |  | 110 | 92 |  |
| Positive | 43 | 82 |  | 73 | 40 |  | 67 | 45 |  |
| **Nervous invasion** |  |  | 0.447 |  |  | 0.833 |  |  | 0.419 |
| Negative | 116 | 201 |  | 194 | 92 |  | 164 | 123 |  |
| Positive | 14 | 18 |  | 20 | 8 |  | 13 | 14 |  |
| **Tumor deposit** |  |  | 0.614 |  |  | 0.464 |  |  | 0.863 |
| ≤2 | 116 | 191 |  | 185 | 90 |  | 154 | 121 |  |
| ＞2 | 14 | 28 |  | 29 | 10 |  | 23 | 16 |  |
| **Colonic perforation** |  |  | 0.774 |  |  | 0.758 |  |  | 0.074 |
| No | 126 | 210 |  | 205 | 97 |  | 167 | 135 |  |
| Yes | 4 | 9 |  | 9 | 3 |  | 10 | 2 |  |
| **Tumor budding** |  |  | ＜0.001 |  |  | 0.013 |  |  | 0.243 |
| ＜5 cells | 37 | 100 |  | 74 | 50 |  | 63 | 58 |  |
| ≥5 cells | 93 | 119 |  | 140 | 50 |  | 114 | 79 |  |
| **Histological type** |  |  | 0.505 |  |  | 0.074 |  |  | 0.739 |
| Adenocarcinoma | 112 | 194 |  | 181 | 92 |  | 152 | 120 |  |
| Mucinous/SRCC | 18 | 25 |  | 33 | 8 |  | 25 | 17 |  |
| **Ulceration** |  |  | 0.654 |  |  | 0.713 |  |  | 0.133 |
| No | 77 | 123 |  | 127 | 57 |  | 111 | 74 |  |
| Yes | 53 | 96 |  | 87 | 43 |  | 66 | 63 |  |
| **Pathological T stage** |  |  | ＜0.001 |  |  | 0.010 |  |  | 0.194 |
| T1-2 | 17 | 66 |  | 41 | 33 |  | 40 | 40 |  |
| T3-4 | 113 | 153 |  | 173 | 67 |  | 137 | 97 |  |
| **LNM** |  |  | 0.072 |  |  | 0.223 |  |  | 0.818 |
| No | 68 | 137 |  | 119 | 63 |  | 104 | 78 |  |
| Yes | 62 | 82 |  | 95 | 37 |  | 73 | 59 |  |
| **TNM stage** |  |  | 0.059 |  |  | ＜0.001 |  |  | 0.733 |
| I+ II | 62 | 128 |  | 111 | 57 |  | 93 | 75 |  |
| III+ IV | 68 | 91 |  | 214 | 43 |  | 84 | 62 |  |
| **sTILs** |  |  |  |  |  | ＜0.001 |  |  | 0.019 |
| Poor | ---- | ---- |  | 98 | 23 |  | 78 | 42 |  |
| Rich | ---- | ---- |  | 116 | 77 |  | 99 | 95 |  |
| **iTILs** |  |  | 0.016 |  |  | 0.281 |  |  | 0.511 |
| Poor | 130 | 210 |  | 206 | 99 |  | 173 | 132 |  |
| Rich | 0 | 10 |  | 8 | 1 |  | 4 | 5 |  |
| **CD3^*^** |  |  | ＜0.001 |  |  |  |  |  | 0.001 |
| Poor | 98 | 116 |  | ---- | ---- |  | 134 | 80 |  |
| Rich | 22 | 78 |  | ---- | ---- |  | 43 | 57 |  |
| **CD20^*^** |  |  | 0.019 |  |  | 0.001 |  |  | ---- |
| Poor | 78 | 99 |  | 134 | 43 |  | ---- | ---- |  |
| Rich | 42 | 95 |  | 80 | 57 |  | ---- | ---- |  |
| ***Schistosomiasis*** |  |  | 0.308 |  |  | 0.219 |  |  | 0.728 |
| Negative | 74 | 138 |  | 123 | 65 |  | 104 | 84 |  |
| Positive | 56 | 81 |  | 91 | 35 |  | 73 | 53 |  |
| ----:Data is not applicable; Abbreviation: sTILs=stromal tumor-infiltrating; N=Number; SRCC= signet ring cell carcinoma; LNM=lymph node metastasis. iTILs=Intratumoral-infiltrating lymphocytes. The association between schistosomiasis and clinicopathological characteristics was evaluated by using the Chi square and Fisher’s exact tests. *=Missing data (although 349 CRC patients were enrolled in the cohort, some sample tissues were unavailable for the immunohistochemical assay, such as those with high fat content. Finally, there were 314 cases could be used for the following analysis of CD3 and CD20). | | | | | | | | | |
|  | | | | | | | | | |
